# Iron Chelation Reduces Intracellular Hydroxyl Radicals in Normal Human Dermal Fibroblasts Independently of Aging

**DOI:** 10.3390/antiox14121437

**Published:** 2025-11-28

**Authors:** Kazunori Takemoto, Ami Ozaki, Yusuke Tanii, Masayuki Yagi, Masamitsu Ichihashi, Hideya Ando

**Affiliations:** 1 Department of Medical Technology, Okayama University of Science, 1-1 Ridai-cho, Kita-ku, Okayama 700-0005, Japan; k-takemoto@ous.ac.jp; 2 Department of Bioscience, Okayama University of Science, 1-1 Ridai-cho, Kita-ku, Okayama 700-0005, Japan; 3 ROSETTE, Co., Ltd., 3-26-10, Higashi-Shinagawa, Shinagawa, Tokyo 140-0002, Japan; 4 Arts Ginza Clinic, 2-20-15 Shinbashi, Minato-ku, Tokyo 105-0004, Japan

**Keywords:** hydroxyl radical, iron accumulation, dermal fibroblast, senescence, iron chelator, hydrogen peroxide

## Abstract

In cultured skin cells, decreases in antioxidant function and increases in intracellular free Fe^2+^ due to replicative aging have been reported. The Fenton reaction between Fe^2+^ and hydrogen peroxide is a threat to the skin because it produces hydroxyl radicals that attack proteins, nucleic acids and lipids. The purpose of this study was to determine whether exogenous iron modulation alters intracellular hydroxyl radicals in senescent normal human dermal fibroblasts (NHDFs). As previously reported, reduced antioxidant function, the accumulation of Fe^2+^ and increased levels of Reactive Oxygen Species (ROS) were observed in senescent NHDFs. The novel catalase (CAT) activity assay demonstrated a decrease in CAT activity alone in aged NHDFs. However, sufficient CAT activity against hydrogen peroxide was still maintained. Young NHDFs showed an increase in intracellular Fe^2+^ and hydroxyl radical signals after exogenous iron supplementation, both of which were cancelled by an iron chelator. Under the same experimental conditions, aged NHDFs that already showed a higher concentration of intracellular Fe^2+^ and stronger hydroxyl radical signals than young NHDFs also elicited a reduction in these levels after the addition of an iron chelator. These results suggest that exogenous regulation of intracellular iron concentration by iron chelators can suppress hydroxyl radical production independently of senescence progression, offering promise for future developments in senescence prevention research.

## 1. Introduction

Cellular aging of skin tissues leads not only to changes in appearance, such as increased numbers of wrinkles and pigmented spots, but also to the functional deterioration of cells that make up the skin. Oxidative stress, a major cause of those changes, has been widely reported to increase with age [1]. In dermal fibroblasts, there are multiple causes of cellular senescence due to a complex interplay of various mechanisms, including nuclear DNA damage [2], generation of excessive ROS, and mitochondrial dysfunction [3,4,5]. Cellular senescence has been reported to cause decreased production of extracellular matrix collagen and elastin [6], increase levels of intracellular metal ions such as iron and zinc [7], and decrease the activity of antioxidant enzymes that scavenge ROS [8]. Although the mitogenic capacity of senescent cells is reduced, their metabolic activity is maintained, and they secrete mixed molecules known as senescence-associated secretory phenotype (SASP) that contribute to inflammation [9], leading to the further proliferation of senescent cells and increased inflammation in surrounding tissues. In vitro, the accumulation of free Fe^2+^ has been found to occur due to replicative senescence of fibroblasts [10]. Free Fe^2+^ catalyzes the formation of oxidants that damage biomacromolecules, such as hydroxyl radicals, via the Fenton reaction with hydrogen peroxide [11,12]. Hydrogen peroxide is produced by a variety of reactions in cells, but in every reaction, it eventually reacts with iron via the Fenton reaction [13]. The increased production of ROS and the induction of ferroptosis via mitochondrial abnormalities [14] have also been reported in iron-rich conditions. In this study, we examined the effects of exogenous iron regulation using iron chelators on intracellular hydroxyl radicals in aged NHDFs, which exhibited increased intracellular free Fe^2+^ and reduced antioxidant function, by comparing them with young NHDFs. Whether iron ion accumulation and the resulting hydroxyl radical production are a cause or consequence of aging remains a subject of ongoing debate. Furthermore, there is a growing need for the development of treatments and drugs targeting early aging prevention and the removal of senescent cells. The findings of this study, which involved externally controlling iron ions, are significant for the foundational fields of skincare, cosmetics, and dermatopharmacology.

## 2. Materials and Methods

### 2.1. Reagents

Hydrogen peroxide, sodium azide (NaN_3_), 3-amino-1H-1,2,4-triazole (3-AT), iron (II) nitrate hexahydrate (Fe(NO_3_)_3_), radioimmunoprecipitation assay (RIPA) buffer, and 2-mercaptoethanol were purchased from FUJIFILM Wako Pure Chemical Corporation (Osaka, Japan). Deforoxamine–mesylate (DFO) was purchased from Cayman Chemical Company (Ann Arbor, MI, USA).

### 2.2. Cell Culture

Cryopreserved primary NHDFs were purchased from Cascade Biologics, LLC (Waltham, MA, USA). NHDFs were cultured in Dulbecco’s Modified Eagle’s Medium (DMEM)-D6046 supplemented with 1% antibiotic anti-mycoplasma solution and 10% fetal bovine serum (FBS) and maintained in a humidified incubator at 37 °C with 5% CO_2_. The culture medium was replaced every 2 or 3 days. When cell confluency reached approximately 90%, NHDFs were dissociated using trypsin. Cell passage numbers were controlled between 3 and 12 as young, 12 and 15 as middle, and 15 and 25 as aged generations. At the time of cell harvest, cells were detached with 0.25% Trypsin-EDTA and washed with phosphate-buffered saline (PBS). Reagents supplied to the cells were prepared via dilution in DMEM-D5921, Eagle’s minimal essential medium (E-MEM), and Hanks’ Balanced Salt Solution (HBSS(+)) to optimize experimental conditions. DMEM-D6046, D5921, and antibiotic anti-mycoplasma solution were purchased from Merck KGaA (Darmstadt, Germany). FBS was purchased from MP Biomedicals (San Diego, CA, USA). E-MEM was purchased from Fujifilm Wako Pure Chemicals Corporation (Osaka, Japan). Trypsin-EDTA, PBS, and HBSS(+) were purchased from Thermo Fisher Scientific (Waltham, MA, USA).

### 2.3. Evaluation of Senescent Cells

In this study, primary cultured NHDFs obtained from neonatal tissues were defined as young (68 ± 15 days) or aged (141 ± 28 days) cells, depending on the duration of culture. Cells cultured for approximately 100 days were defined as middle (Table 1). Twenty-four h after cell seeding at 5000 cells/cm^2^, cells were stained according to the standard protocol of the SA-β-galactosidase staining kit (Cell Biolabos, Inc., San Diego, CA, USA). Cells that were confirmed to stain positive for SA-β-galactosidase using a microscope were determined to be senescent cells. After staining, cells were observed and recorded with a Cell Sens Dimension microscope system (Olympus Corporation., Tokyo, Japan), and cell number and size were determined from multiple recorded images using a FLOVEL filing system (FLOVEL Corporation., Kanagawa, Japan). Next, 1 × 10^5^ cells were seeded into a 6-well plate. The aging level was evaluated based on cell doubling time using the cell count after 108 h of culture from seeding. Cell doubling time was calculated using the following equation: Cell doubling time = 3.32 × (log Nf/log Ni)/culture time (108 h), where Nf represents the cells at harvest and Ni represents the seeded cells at the start of culture.

### 2.4. Measurement of Catalase Activity

Cell pellets were lysed in RIPA Buffer containing 1% protease inhibitor cocktail (Merck KGaA). The supernatant obtained after centrifugation (15,000 rcf, 3 min) was used as the cell lysate. Cell lysates were frozen at −20 °C until use in subsequent experiments after protein concentrations were determined with the Pierce^TM^ BCA Protein Assay Kit (Thermo Fisher Scientific). CAT activity was determined from the cell lysates according to a previously reported method utilizing CAT inhibitors [15]. Specifically, 10 μg of total protein (final concentration 0.04 μg/μL) was reacted at room temperature for 30 min with a mixture of a CAT inhibitor adjusted in PBS (pH 7.4) and 50 μM hydrogen peroxide, and the residual hydrogen peroxide was measured. CAT activity in young and aged samples was converted to the activity of commercially available bovine liver catalase (9000 units/mg) for comparison.

### 2.5. Measurement of Hydrogen Peroxide Production

After washing the medium of NHDFs cultured at 5 × 10^4^/cm^2^ with PBS, it was replaced with FBS-free E-MEM medium with NaN_3_ (1 mM) or 3-AT (25 mM) added to inhibit hydrogen peroxide removal by CAT. After 24 h of incubation, the hydrogen peroxide concentration in the culture medium was measured using the OxiSelect™ Hydrogen Peroxide Assay Kit (Cell Bio Labs., Inc., San Diego, CA, USA). Following the standard protocol, the fluorescence of resorufin, which is produced by the reaction of hydrogen peroxide and 10-acetyl-3,7-dihydroxyphenoxazine (ADHP) in the presence of peroxidase, was measured. The fluorescence was measured (Ex/Em = 550/610 nm) using a Micro Plate Reader MTP-900 (Corona Electric., Ibaraki, Japan).

### 2.6. Western Blotting

First, 1 × 10^5^ cell pellets were lysed in RIPA Buffer containing 1% protease inhibitor cocktail. The supernatant obtained after centrifugation (15,000 rcf, 3 min) was analyzed for protein concentration using the Pierce™ BCA Protein Assay Kit (Thermo Fisher Scientific). After dilution with sample buffer containing 2-mercaptoethanol, the mixture was heated at 85 °C for 5 min. A sample containing 15 μg of protein per lane was separated on a polyacrylamide gel (constant current 25 mA, 60 min) and then blotted onto a polyvinylidene difluoride (PVDF) membrane using the Trans Blot Turbo system (BIO-RAD) (2.5 A, 25 V, 7 min). The PVDF membrane with transferred proteins was blocked at room temperature for 30 min using wash buffer containing 3% bovine serum albumin—BSA (Nacalai Tesque, Inc., Kyoto, Japan). The primary antibodies used, Catalase(H-9):sc-271803, GPx4(B-12):sc-166120, β-actin(C4):sc-47778, Lamin B1(B-10):sc-374015, and p21(F-5):sc-6246, were reacted at 1:2000 dilution for 60 min at room temperature or overnight at 4 °C. FTL (F4T8H) Rabbit mAb #68106 was reacted at a 1:2000 dilution for 60 min at room temperature. 4-Hydroxynonenal (4-HNE) Antibodies (Thermo Fisher Scientific) were reacted at a 1:1000 dilution for 2 h at room temperature. These were reacted with m-IgGK BP-HRP:sc-516102 or Anti-Rabbit IgG, with the HRP-linked Antibody #7074 as the secondary antibody, at room temperature for 60 min at 1:2000 dilution. Luminescence was detected using Clarity^TM^ Western ECL Substrate with ChemiDoc^TM^ XRS+ and Image Lab^TM^ Software 6.1 (Bio-Rad Laboratories Inc., Hercules, CA, USA). Related equipment, Laemmli Sample Buffer, acrylamide gel, PVDF membrane, Tris/Glycine/SDS Buffer, Tween 20, TBS wash buffer, and Clarity™ Western ECL Substrate were purchased from Bio-Rad. The antibodies used were purchased from Santa Cruz Biotechnology, Inc (Dallas, TX, USA). or Cell Signaling Technology, Inc (Danvers, MA, USA).

### 2.7. Real-Time Polymerase Chain Reaction (RT-PCR)

Total RNA was extracted from cell pellets using a FavorPrep Blood/Cultured Total RNA Mini Kit (Favogen Biotech Corp., Ping Tung, Taiwan). cDNA was synthesized from 2 μg of total RNA following the standard protocol for PrimeScript™ IV 1st Strand cDNA Synthesis Mix (Takara Bio Inc., Shiga, Japan). Using 0.2 μg of this cDNA as template, quantitative real-time PCR was performed following the standard protocol for THUNDERBIRD^®^ SYBR™ qPCR Mix (Toyobo Inc. Oaska, Japan). RT-PCR was run on a Step One Plus^TM^ Real-Time PCR System Upgrade (Thermo Fisher Scientific). The mRNA expression levels obtained by means of PCR were compared to each other relative to the mRNA expression level of young NHDFs. The primer sequences used for RT-PCR are shown in Table 2. Synthesis of these primers was outsourced to Thermo Fisher Scientific’s custom oligonucleotide synthesis service.

### 2.8. Fluorescent Staining (Superoxide, Fe^2+^, HPF, and Mitochondria)

After washing the medium in which the NHDFs were cultured at 5 × 10^4^/cm^2^ with PBS, staining was performed using various fluorescent staining kits. Superoxide detection was performed by adding MitoSox™ Red mitochondrial superoxide indicator (Thermo Fisher Scientific), prepared at 1.0 μM in HBSS(+), to cells and incubating at 37 °C for 30 min. To detect intracellular free Fe^2+^, cells were stained with the Ferro Orange staining kit (DOJINDO LABORATORIES., Kumamoto, Japan) prepared at 1.0 μM in HBSS(+) for 30 min at 37 °C. To detect intracellular hydroxyl radicals, cells were stained with Hydroxyphenyl Fluorescein (HPF staining kit, Goryo Chemical., Sapporo, Japan) prepared at 50 μM in HBSS(+) for 30 min at 37 °C. To detect free Fe^2+^ within mitochondria, the MitoFerroGreen staining kit (DOJINDO LABORATORIES) was prepared to a concentration of 20 μM in HBSS(+) and used for staining at 37 °C for 30 min. For mitochondrial staining, Rhodamine 123 (FUJIFILM Wako Pure Chemical Corporation., Osaka, Japan) or MitoTracker^®^ Deep Red FM #8778 (Cell Signaling Technology, Inc. (Danvers, MA, USA) was prepared to a concentration of 1.0 μM in HBSS(+) and incubated at 37 °C for 30 min. After three washes with PBS, Hoechst 33342, prepared to 2.0 μM in HBSS(+), was added to the cells, and nuclear staining was performed at 37 °C for 10 min. Staining was performed according to the respective standard protocols. Cells were observed and recorded using the Cell Sens Dimension microscope system. Fluorescence intensity was measured from multiple recorded images using the luminance measurement mode of the FLOVEL Filing System.

### 2.9. Image Measurement and Image Adjustment

Using the luminance measurement mode of the FLOVEL Filing System, fluorescence intensity was measured from multiple unedited images. The contrast settings, brightness, and luminance adjustments applied to the images presented in this paper were uniformly applied across the entire image area within each section using the Photos application version 10 (Apple Inc., Cupertino, CA, USA).

### 2.10. Cellular Exposure to DFO and Iron or Hydrogen Peroxide

DFO was adjusted to a 100 mM stock solution in purified distilled water and was stored frozen at −20 °C until use [16]. These supplements were added to FBS-free E-MEM to achieve a concentration of 100 μM (or the desired concentration) and added to the cells. Cells were exposed to 50μM hydrogen peroxide diluted in E-MEM for each use. Dose-dependent cytotoxicity induced by DFO and Fe(NO_3_)_3_ was examined with the CellTiter 96^®^ AQueous One Solution Cell Proliferation (MTS) Assay (Promega Corp., Madison, WI, USA).

### 2.11. Hydroxyl Radical Induction by Hydrogen Peroxide

Young NHDFs were seeded at a density of 5000 cells/cm^2^. After 24 h, they were washed with PBS and then exposed for 24 h to FBS-free DMEM-D5921, containing 0, 5, or 10 μM hydrogen peroxide. Cells were washed three times with PBS after removing the medium, and hydrogen peroxide-induced hydroxyl radicals were detected using HPF fluorescent staining. The fluorescence intensity measured from the stained cells was compared according to the concentration of hydrogen peroxide to which they were exposed.

### 2.12. Statistical Evaluation

Data are shown as the mean ± standard deviation or as box-and-whisker plots with quartiles. Comparisons among multiple groups were performed using analysis of variance (ANOVA) followed by Tukey’s multiple comparison test. Paired samples were analyzed using Student’s *t*-test. A significance level of *p* < 0.05 was considered statistically significant. Statistical analysis and graph creation were performed using Microsoft Excel version 16.103.2. (Microsoft Corp., Redmond, WA, USA).

## 3. Results

### 3.1. The Number of Senescent NHDFs Positive for SA-β-Galactosidase Increased with Increasing Time in Culture

**Figure 1:**
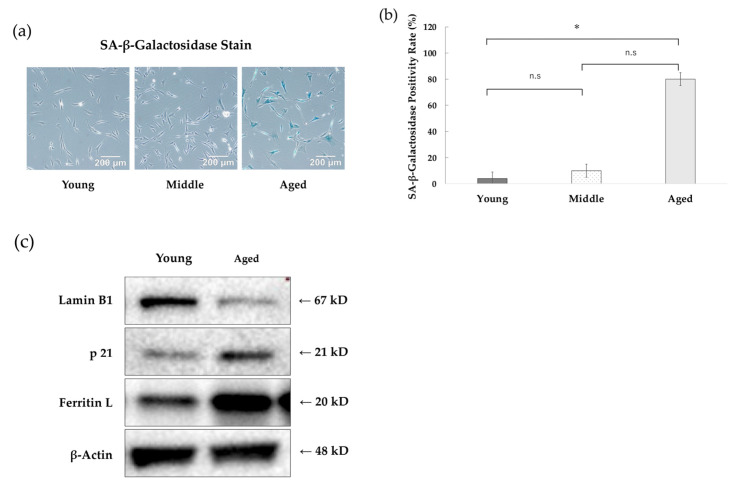
Classification of Senescent Stages. (**a**) The blue-stained SA-β-galactosidase-positive cells were determined to be senescent cells. SA-β-galactosidase staining was performed independently 5 times in each cell population (*n* = 5). At least five fields of view were observed. Representative images are shown. (**b**) SA-β-Galactosidase staining positivity rate. The SA-β-galactosidase positivity rate was calculated for cells observed in (**a**). The mean and standard deviation (*n* = 5) are shown. One-way ANOVA and Tukey’s multiple comparison test were performed. * indicates a *p*-value < 0.05 and was considered statistically significant. (**c**) Expression of aging markers. Western blot analysis of Lamin B1, p21 protein, ferritin L chain, and β-actin—markers of aging—was performed on lysates from young and aged NHDFs.

NHDFs cultured for 68 ± 15 days after the start of primary culture were defined as young, those cultured for around 100 days were defined as middle, and those cultured for 141 ± 28 days were defined as aged (Table 1). The cell doubling time in young and aged NHDFs was 12.6 ± 0.9 h and 23.7 ± 4.2 h, respectively (*n* = 3, *p* < 0.05, Student’s *t*-test). When those cells were stained for SA-β-galactosidase, an indicator of senescence, few young NHDFs were observed to be stained for SA-β-galactosidase. In contrast, middle NHDFs were weakly stained, while aged NHDFs were more strongly stained (Figure 1a). Calculating the SA-β-galactosidase staining positivity rate revealed a clear increase in the aged NHDFs (Figure 1b). The aged NHDFs showed a greater number of hypertrophied cells, a morphological feature of cellular aging, compared to the young NHDFs. Furthermore, Western blot analysis revealed that compared to the young NHDFs, the aged NHDFs exhibited increased p21 protein and ferritin L accumulation, along with decreased Lamin B1 (Figure 1c). These results demonstrated that aged NHDFs are in a replicative senescence state.

### 3.2. Decreased Expression of CAT and GPx4 and Decreased CAT Activity with Replicative Aging of NHDFs

**Figure 2:**
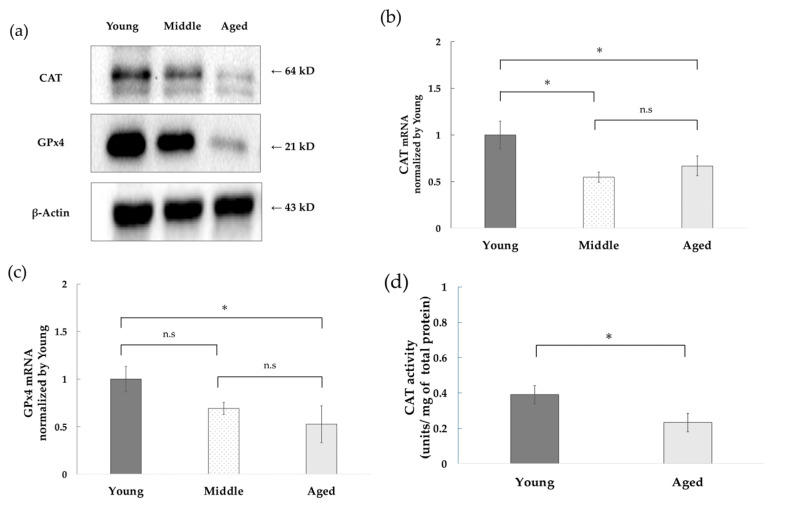
Decline in antioxidant function of NHDFs with aging. (**a**) Decreased antioxidant protein expression with aging. Proteins extracted from young, middle, and aged NHDFs were analyzed via Western blot. Of note, 15 μg of protein was loaded per lane. (**b**) Decrease in CAT mRNA expression due to aging. CAT mRNA expression in young, middle, and aged NHDFs was analyzed via quantitative real-time PCR (triplicate) using known DNA concentrations (*n* = 3). The mean and standard deviation are shown. CAT mRNA expression levels were normalized to young NHDF expression levels for relative comparison. (**c**) Decrease in GPx4 mRNA expression due to aging. GPx4 mRNA expression in young, middle, and aged NHDFs was analyzed via quantitative real-time PCR (triplicate) using known DNA concentrations (*n* = 3). The mean and standard deviation are shown. GPx4 mRNA expression levels were normalized to young NHDF expression levels for relative comparison. (**d**) The amount of hydrogen peroxide removed per 10 μg of total protein (final concentration 0.04 μg/μL) was measured in young and aged NHDFs and converted to bovine liver catalase activity (*n* = 5). Data are shown as the mean ± standard deviation and compared between the young and aged NHDFs. For (**b**,**c**), one-way ANOVA and Tukey’s multiple comparison test were performed. For (**d**), Student’s *t*-test was performed. A *p*-value < 0.05 was considered statistically significant. * indicates *p* < 0.05, while n.s indicates no significant difference.

To investigate the antioxidant function of young and aged NHDFs, the expression of CAT and GPx4, which are involved in hydrogen peroxide removal, was examined via Western blotting. Protein expression levels of CAT and GPx4 decreased as the culture duration of NHDFs increased (Figure 2a). Quantitative RT-PCR analysis of CAT and GPx4 mRNA expression demonstrated a significant decrease in mRNA expression levels for both genes with increasing NHDF culture days, consistent with the Western blot results (Figure 2b,c). However, no differences in β-actin mRNA expression levels were observed between aging stages at this time point (Supplementary Material S1). Measurement of CAT activity per 10 μg of total protein extracted from cells (0.04 μg/μL) revealed a significant decrease in aged cells (0.23 ± 0.05 units/mg of total protein) compared to young cells (0.39 ± 0.05 units/mg of total protein) (Figure 2d). These results suggest a decline in antioxidant capacity associated with replicative senescence in cultured cells.

### 3.3. Intracellular Free Fe^2+^ Accumulates and ROS Increases in Senescent NHDFs

**Figure 3:**
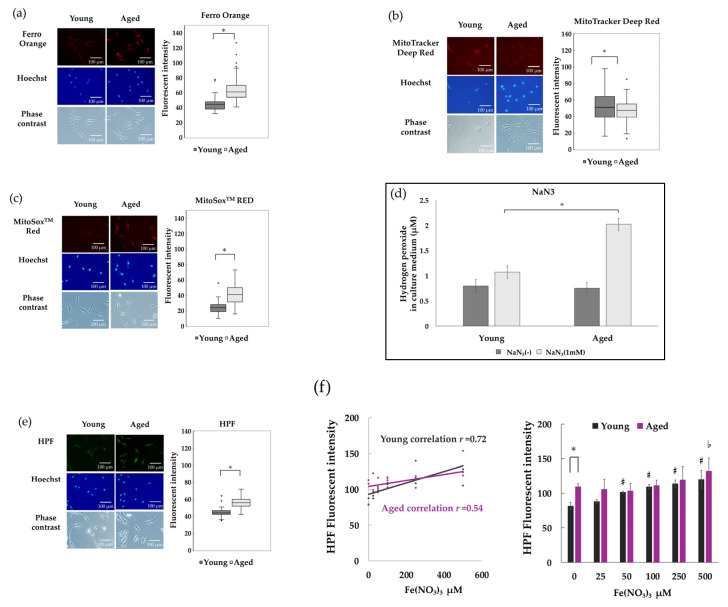
Iron accumulation and increased ROS due to replicative senescence of NHDFs. (**a**) Intracellular free Fe^2+^ fluorescence staining images and comparison of free Fe^2+^ fluorescence intensity. Stained cells were observed in multiple fields of view, and representative images are shown. The scale bar indicates 100 μm. The fluorescence intensities of young NHDFs (80 cells) and aged NHDFs (82 cells) obtained from three independent staining experiments (*n* = 3) were compared using box-and-whisker plots. (**b**) Mitochondrial decline in aged NHDFs. Multiple fields of view of NHDF cells stained with Mito Tracker Deep Red were observed. Representative images are shown. The scale bar indicates 100 μm. The fluorescence intensities of young (81 cells) and aged (68 cells) NHDFs obtained from three independent experiments (*n* = 3) were compared using box-and-whisker plots. (**c**) Increase in intracellular superoxide in aged NHDFs. Stained NHDFs were observed from multiple viewpoints, and representative images are shown. The scale bar indicates 100 μm. The fluorescence intensities of young (50 cells) and aged (73 cells) NHDFs obtained from three independent staining experiments (*n* = 3) were compared using box-and-whisker plots. (**d**) Hydrogen peroxide release from NHDFs inhibited by NaN_3_. Hydrogen peroxide concentrations secreted into the culture medium over 24 h from young and aged NHDFs were measured with or without CAT inhibition. CAT was inhibited by NaN_3_ (1.0 mM). Four independent measurements were performed (*n* = 4), and the mean ± standard deviation is shown. (**e**) Intracellular HPF fluorescence staining image and comparison of HPF fluorescence intensity. Stained NHDFs were observed from multiple viewpoints, and representative images are shown. The scale bar indicates 100 μm. The fluorescence intensities of young (56 cells) and aged (56 cells) NHDFs obtained from three independent staining experiments (*n* = 3) were compared using box-and-whisker plots. The boxes with quartiles shown in each section represent the median, 25th percentile, and 75th percentile. Whiskers indicate the maximum and minimum values within 1.5 times the interquartile range. Outliers are plotted separately. Dark boxes represent the young NHDFs, while light boxes represent the aged NHDFs. A Student’s *t*-test was performed, and a significance level of *p* < 0.05 was considered statistically significant. * indicates *p* < 0.05, while n.s indicates no significant difference. (**f**) The left panel shows a positive correlation between HPF fluorescence intensity in young (dark line and dots) and aged (pink line and dots) NHDFs and Fe(NO_3_)_3_ dose. The right panel shows the HPF fluorescence intensity in young (dark bars) and aged (pink bars) NHDFs as the mean and standard deviation for each Fe(NO_3_)_3_ dose. ANOVA and Tukey’s multiple comparison analysis were performed, with *p* < 0.05 indicating significant differences. * indicates *p* < 0.05. # indicates *p* < 0.05 vs. 0 μM. ♭ indicates *p* < 0.05 vs. 0 μM.

Intracellular free Fe^2+^ in NHDFs was stained with Ferro Orange, and the fluorescence intensity of young NHDFs and aged NHDFs was compared. Aged NHDFs exhibited significantly higher fluorescence intensity than young NHDFs, suggesting that free Fe^2+^ accumulates in senescent cells (Figure 3a). It is known that human fibroblast aging induces the accumulation of free Fe^2+^ within mitochondria [10]. We measured mitochondrial membrane potential and free Fe^2+^ accumulation in young and aged NHDFs, but found no difference in membrane potential and free Fe^2+^ within mitochondria (Supplementary Materials S2 and S3). However, measurement of mitochondrial mass revealed significantly lower signal in aged NHDF compared to young NHDF, suggesting a reduction in mitochondria (Figure 3b). Next, we examined superoxide production from mitochondria in NHDFs using MitoSox™ Red. Superoxide signals were significantly increased in aged NHDFs compared to young NHDFs (Figure 3c). Without CAT inhibition, hydrogen peroxide in the culture medium was almost completely removed regardless of culture duration for both aged and young NHDFs (Figure 3d, Supplementary Material S4). Hydrogen peroxide released from CAT-inhibited NHDFs was significantly increased in the aged. Furthermore, HPF intensity, indicative of intracellular hydroxyl radicals, showed a significant increase in aged NHDFs compared to young NHDFs (Figure 3e). Correlation analysis between iron dose and HPF fluorescence intensity revealed a positive correlation in young NHDFs, suggesting an iron dose-dependent increase in HPF. However, this correlation was not observed in aged NHDFs (Figure 3f left). Without iron supplementation, the HPF intensity of aged NHDFs was already significantly higher than that of young NHDFs and showed no significant response to iron doses up to 500 μM compared to the unsupplemented control (Figure 3f right).

### 3.4. Hydroxyl Radicals in NHDFs Induced by Hydrogen Peroxide and Fe^2+^ Were Attenuated by Iron Chelators

**Figure 4:**
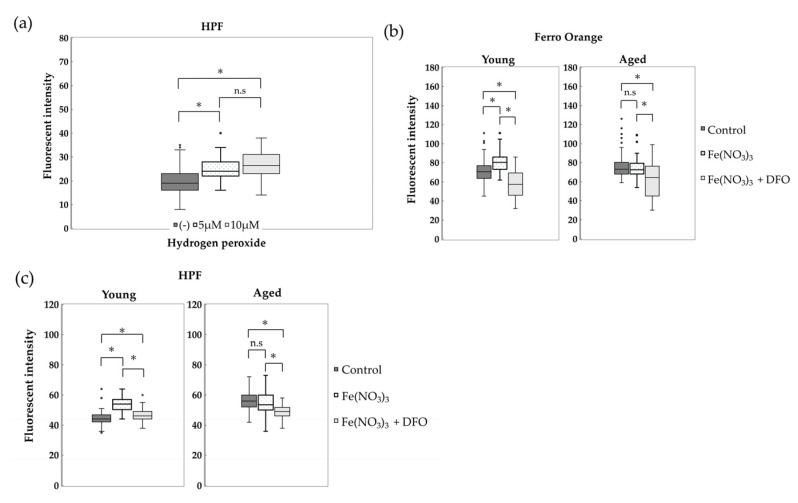
Induction of hydroxyl radicals by hydrogen peroxide and its suppression by iron chelators. (**a**) HPF fluorescence intensity was measured independently three times in young NHDFs exposed to E-MEM supplemented with 0, 5, or 10 μM hydrogen peroxide for 24 h. Fluorescence intensity by hydrogen peroxide concentration is shown in box plots with quartiles. The number of cells measured for hydrogen peroxide concentrations of 0, 5, and 10 μM was 40 cells each. (**b**) Comparison of Ferro Orange fluorescence in young and aged NHDFs. Fluorescence intensity was measured in young and aged NHDFs from the control group, Fe(NO_3_)_3_ group, and Fe(NO_3_)_3_ + DFO group (114 cells per group) (*n* = 5) and compared using a box plot. (**c**) Comparison of HPF fluorescence in young and aged NHDFs. Fluorescence intensity was measured in young and aged NHDFs from the control group, Fe(NO_3_)_3_ group, and Fe(NO_3_)_3_ + DFO group (56 cells per group) (*n* = 3) and compared using a box plot. In each section, the dark boxes indicate the control group, the dotted boxes indicate the Fe(NO_3_)_3_ group, and the light boxes indicate the Fe(NO_3_)_3_ + DFO group. The boxes with quartiles shown in each section represent the median, 25th percentile, and 75th percentile. The whiskers indicate the upper and lower limits of the maximum values within 1.5 times the interquartile range. Outliers are plotted individually. One-way ANOVA and Tukey’s multiple comparison test were performed. A significance level of *p* < 0.05 was considered statistically significant. * indicates *p* < 0.05, and n.s indicates no significant difference.

To investigate hydrogen peroxide-induced hydroxyl radical generation, young NHDF cells were exposed for 24 h at 37 °C under 5% CO_2_ in FBS-free E-MEM supplemented with hydrogen peroxide (0, 5, 10 μM). HPF fluorescence intensity, an indicator of intracellular hydroxyl radicals, increased in a hydrogen peroxide dose-dependent manner (Figure 4a). Prior to iron and DFO stimulation of NHDFs, we examined the cytotoxicity of iron and DFO doses. NHDFs showed no significant cytotoxicity at Fe(NO_3_)_3_ and DFO concentrations ranging from 0 to 500 μM (Supplementary Materials S5 and S6). Ferro Orange fluorescence intensity, indicating intracellular free Fe^2+^ in NHDFs, was measured after 24 h exposure to E-MEM supplemented with Fe(NO_3_)_3_. In young NHDFs, Ferro Orange fluorescence intensity significantly increased, whereas in aged NHDFs, the Ferro Orange signal did not increase. Exposure of NHDFs to E-MEM containing a mixture of Fe(NO_3_)_3_ and DFO resulted in a marked decrease in the Ferro Orange signal in both young and aged NHDFs (Figure 4b). On the other hand, examination of HPF fluorescence indicating hydroxyl radicals in NHDFs exposed to E-MEM supplemented with Fe(NO_3_)_3_ revealed a significant increase in HPF signal in young NHDFs. However, the HPF signal did not increase in aged NHDFs. In NHDFs exposed to E-MEM containing a mixture of Fe(NO_3_)_3_ and DFO, the HPF fluorescence signal was markedly reduced in both young and aged NHDFs (Figure 4c). These results suggest that hydrogen peroxide and intracellular free Fe^2+^ induce hydroxyl radical generation and that the supply of iron chelators suppresses hydroxyl radical generation in NHDFs. Furthermore, it was suggested that the suppression of hydroxyl radicals by iron chelators occurs independently of the level of cellular senescence.

## 4. Discussion

Aged NHDFs exhibiting replicative aging showed reduced antioxidant function, characterized by decreased CAT and GPx4 expression as determined via Western blot analysis and quantitative real-time PCR. Furthermore, a decline in pure CAT activity due to aging was also demonstrated. The hydrogen peroxide removal activity currently widely regarded as CAT activity is a composite effect of multiple hydrogen peroxide scavengers and cannot be considered true CAT activity. Using a novel method involving CAT inhibitors [15], we measured the activity of pure CAT alone for the first time and demonstrated that the activity of the single CAT enzyme declines with aging. Furthermore, under CAT-inhibited conditions, the amount of hydrogen peroxide released into the culture medium from aged NHDFs increased compared to young NHDFs. Meanwhile, aged NHDF showed accumulation of free Fe^2+^ and increased hydroxyl radicals, both of which were mitigated by DFO. Notably, the increase in hydroxyl radicals induced by iron and hydrogen peroxide was observed independently of cellular senescence, and the reduction in hydroxyl radicals by DFO was also not dependent on cellular senescence. The decline in antioxidant function associated with NHDF aging was consistent with previous reports [8,10]. This strongly supports the view that aged cells are exposed to stronger oxidative stress than young cells, indicating that aged cells are more susceptible to the effects of residual hydrogen peroxide in surrounding tissues. In human skin fibroblasts, the expression of CAT and Gpx decreases with aging [17]. Other antioxidant proteins, such as heme oxygenase-1, are no longer induced with aging [18], and quinone oxidoreductase shows a decrease in induction with aging [19]. These findings have been reported to be explained by the age-related decline in the Nrf2/ARE signaling pathway, which regulates antioxidant protein expression [20]. In this study, we also observed decreased CAT and GPx4 protein and mRNA expression, along with an age-related decline in CAT activity. These results suggest that the decrease in CAT activity is caused by a quantitative reduction in CAT, implying a potential reduction in Nrf2/ARE signaling. However, this study did not evaluate Nrf2/ARE signaling in aged NHDFs, and thus, no meaningful discussion on this point could be provided.

An increase in intracellular free iron has been reported as a physiological aging response [21]. Aging cells accumulate iron because they do not undergo cell division and thus do not partition iron to daughter cells [22]. A hypothesis has also been proposed that aging-related oxidative stress disrupts iron sensing and uptake, inducing an “iron deficiency” phenotype in cells [10]. Therefore, the increased iron observed in aged NHDFs was interpreted as physiological iron accumulation due to replicative senescence [23,24]. Although increased ferritin was observed in aged NHDFs, lysosomal dysfunction in aged cells could cause ferritin accumulation [25]. It is well known that hydroxyl radicals are generated via the Fenton reaction between Fe^2+^ and hydrogen peroxide [12,13]. An increase in hydroxyl radicals was also observed in aged NHDFs, while a hydrogen peroxide or iron-concentration-dependent increase in hydroxyl radicals was observed in young NHDFs. It is clear that hydrogen peroxide and iron are the cause of the increase in hydroxyl radicals [26]. The fluorescence of HPF, which indicates hydroxyl radicals, overlaps with the fluorescence of lipofuscin, which increases with cellular aging. However, no fluorescence was observed in cells without the staining substrate (Supplementary Material S7). Lipofuscin incorporates iron and contributes to the Fenton reaction, which is known to be significantly inhibited by DFO [27]. DFO has been shown to suppress hydroxyl radical generation via the Fenton reaction and reduce DNA damage [28]. These findings are consistent with the reduction in hydroxyl radicals in DFO-treated NHDF cells, suggesting an antioxidant effect of iron chelation. While a decrease in ROS levels following iron chelation therapy in senescent cells has been previously reported [22], we have now demonstrated that this phenomenon occurs regardless of the degree of cellular senescence.

According to previous reports, superoxide radicals and hydrogen peroxide derived from dysfunctional mitochondria increase unstable iron, leading to further oxidative stress [29,30]. Studies examining mitochondrial function in replicative senescent human fibroblasts have reported that mitochondrial function declines after cellular senescence is complete [31]. Our results showed that mitochondrial mass decreased in aged NHDFs, but no reduction in membrane potential was observed (Supplementary Material S2). This suggests that mitochondrial function was maintained. At this stage, iron levels in aged NHDFs had already increased, indicating impaired antioxidant function and elevated mitochondrial ROS. Our results make it difficult to conclude that mitochondrial dysfunction causes iron accumulation or oxidative stress. Conversely, they support the theory that mitochondrial function is maintained until the completion of replicative senescence. Mitochondria also function as sites of heme synthesis, and heme levels decrease with aging [32,33]. Age-related iron accumulation has been shown to affect ROS production and heme synthesis within mitochondria [34]. Since CAT is a heme protein [35], some correlation between iron accumulation and decreased CAT activity is expected. Although a decrease in CAT activity was observed in aged NHDFs, the amount of hydrogen peroxide in the culture medium was equivalent to that in young NHDFs. No excessive increase in hydroxyl radicals was observed even in aged NHDFs exposed to Fe(NO_3_)_3_. This suggests that the NHDFs we used were in a pre-senescent stage, retaining sufficient defense against hydrogen peroxide despite showing reduced catalase activity. In acatalasemia, CAT activity is almost completely lost, leading to oxidative stress damage [36]. The mechanisms underlying mitochondrial dysfunction and the loss of antioxidant capacity during aging are not yet fully understood and require further investigation.

Diseases reported to involve iron accumulation include Alzheimer’s disease [37], Parkinson’s disease [38,39], diabetes and related kidney diseases [40,41], cardiovascular disease [42], and vitiligo [43]. In skin following UV irradiation, increased abnormal degradation of ferritin by hydrolytic enzymes from damaged lysosomes leads to the release of free Fe^2+^. In senescent cells, an environment exists where levels of unstable iron (oxidized Fe^2+^) increases significantly, in addition to the increase levels of ferritin-bound iron (inactive Fe^3+^) [22]. Therefore, cellular senescence can be predicted to contribute to diseases involving iron accumulation. Removing senescent cells in mice delayed tumor formation and suppressed age-related degeneration in multiple organs, including the kidneys, heart, and adipose tissue [44]. This suggests that suppressing the accumulation of senescent cells has a tissue-protective effect. It is highly intriguing whether the increase in hydroxyl radicals caused by free Fe^2+^ and hydrogen peroxide within senescent cells occurs as tissue damage or as a mechanism for senescent cell removal. In aged NHDFs, 4-hydroxynonenal (4-HNE), which is a lipid peroxidation product indicative of ferroptosis [45], was detected at low levels regardless of elastin (a ferroptosis inducer) addition and was not eliminated by ferrostatin-1 (a ferroptosis inhibitor) (Supplementary Material S8). No iron-dose-dependent cell death occurred (Supplementary Material S5). Therefore, while it is certain that some change in iron metabolism occurs in aged NHDFs, this study could not evaluate whether it stems from aging, oxidative stress, ferroptosis abnormalities, or other causes. This remains a topic for future research. Although ferroptosis is not well characterized, increased intracellular free Fe^2+^, decreased GPx4 [46], and increased hydroxyl radicals causing lipid peroxidation are conditions for ferroptosis occurrence. Induction of ferroptosis by hydroxyl radicals via GPx4 expression inhibition has also been reported [47]. We are currently investigating protein expression related to iron metabolism in NHDFs. It has been demonstrated that senescent cells exhibit resistance to ferroptosis [25], but further investigation is needed to elucidate the mechanism.

Although aged NHDFs released more hydrogen peroxide into the culture medium than young cells, the hydrogen peroxide transport function of the cell membrane is also expected to be related to aging. Aquaporins are proteins belonging to the water channel family, possessing the function of transporting water, glycerol, and H_2_O_2_, and have been reported to regulate cellular functions in skin cells [48,49]. In human dermal fibroblasts, AQP8-mediated H_2_O_2_ efflux into the cytoplasm was shown to function as a defense mechanism against accelerated aging [50]. Future research is needed to clarify whether AQP8 degrades due to aging or oxidative stress.

## 5. Conclusions

This study demonstrated that while antioxidant function declines with aging in NHFD cells, catalase activity against hydrogen peroxide is sufficiently maintained. It is clear that senescent cells exhibit abnormalities in iron metabolism and are exposed to oxidative stress. Whether this is a consequence of senescence or a cause accelerating it remains a subject of ongoing discussion. We confirmed that controlling free Fe^2+^ with iron chelators suppresses hydroxyl radical generation regardless of the cell’s aging level. This suggests potential antioxidant strategies during youth. While further research is needed to fully elucidate aging, oxidative stress, and iron metabolism in skin cells, this study provides fundamental insights relevant to maintaining skin health, skincare, and cosmetic development.

## Figures and Tables

**Table 1:** Classification according to the number of days of cell culture.

Group	Culture Duration (Days)
Young	68 ± 15
Aged	141 ± 28
Middle	Approximately 100 days

NHDFs cultured for 68 ± 15 days from the start of culture to the experiment were defined as young, and NHDFs cultured for 141 ± 28 days were defined as aged, using the start date of culture of purchased primary NHDFs as the first day of culture. NHDFs cultured for approximately 100 days were defined as middle.

**Table 2:** Real-Time PCR Primers.

Primar Name	Sequence	Accession No.
CAT F	GACTGACCAGGGCATCAAAAACC	NM_001752.4
CAT R	TGCCTGATTAAATGTCATGACCTGG	NM_001752.4
Gpx4 F	CGATACGCTGAGTGTGGTTT	NM_001367832.1
Gpx4 R	CGGCGAACTCTTTGATCTCTT	NM_001367832.1
β-actin F	AGAAAATCTGGCACCACACC	NM_001101.5
β-actin F	AGAGGCGTACAGGGATAGCA	NM_001101.5

CAT: Catalase, Gpx4: Glutathione Peroxidase 4.

## Data Availability

The data presented in this study are available from the corresponding author upon request.

## Supplementary Materials

The following supporting information can be downloaded at: https://www.mdpi.com/article/10.3390/antiox14121437/s1, Supplementary Materials S1. β-Actin mRNA expression of NHDFs; Supplementary Materials S2. Mitochondrial membrane potential in NHDFs stained with Rhodamine 123; Supplementary Materials S3. Free Fe2+ in Mitochondria of NHDFs; Supplementary Materials S4. Hydrogen peroxide release from NHDFs inhibited by 3-AT; Supplementary Materials S5. Fe(NO3)3-induced toxicity in NHDFs; Supplementary Materials S6. DFO-induced toxicity in NHDFs; Supplementary Materials S7. Negation of lipofuscin autofluorescence by unstained HPF; Supplementary Materials S8. Detection of 4-HNE indicating lipid peroxidation.

## Abbreviations

The following abbreviations are used in this manuscript:

ADHP	10-acetyl-3,7-dihydroxyphenoxazine
ANOVA	Analysis of variance
BSA	Bovine serum albumin
CAT	Catalase
DFO	Deforoxamine–mesylate
DMEM	Dulbecco’s modified Eagle’s medium
FBS	Fetal bovine serum
E-MEM	Eagle’s minimal essential medium
Fe(NO_3_)_3_	Iron (II) nitrate hexahydrate
GPx4	Glutathione Peroxidase 4
HBSS	Hanks’ Balanced Salt Solution
HPF	Hydroxyphenyl Fluorescein
NaN_3_	Sodium azide
NHDFs	Normal human dermal fibroblasts
Nrf2-ARE	Nuclear factor erythroid 2-related factor 2—Antioxidant Response Element
PBS	Phosphate-buffered saline
PVDF	Poly Vinylidene Di-Fluoride
RIPA	Radio-immune precipitation assay
ROS	Reactive Oxygen Species
RT-PCR	Real-time Polymerase Chain Reaction
SASP	Senescence-associated secretory phenotype
3-AT	3-amino-1H-1,2,4-triazole
4-HNE	4-hydroxynonenal
