# Supplementary material for: Iron Chelation Reduces Intracellular Hydroxyl Radicals in Normal Human Dermal Fibroblasts Independently of Aging
Source: Antioxidants (Basel). 2025 Nov 28;14(12):1437. doi: 10.3390/antiox14121437 (PMC12729569; doi:10.3390/antiox14121437)
Supplement: Supplementary file 1 [file antioxidants-14-01437-s001.zip › antioxidants-3922718-supplementary.pdf]

Supplementary Materials

Supplementary Materials. 1

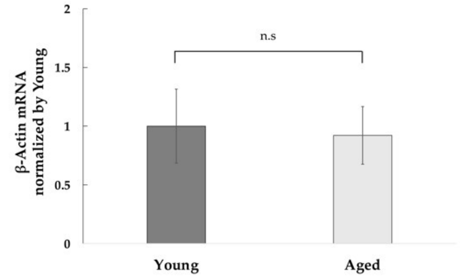

Supplementary Materials.1

β-Actin mRNA expression of NHDFs. β-Actin mRNA expression in Young and Aged NHDFs was analyzed by quantitative real-time PCR (triplicate) using known DNA concentrations of β-Actin (n=3). The mean and standard deviation are shown. β-actin mRNA expression levels were normalized to Young NHDFs expression levels and compared with Aged. Student's t-test was performed, and $p < 0.05$ was considered statistically significant.

Supplementary Materials. 2

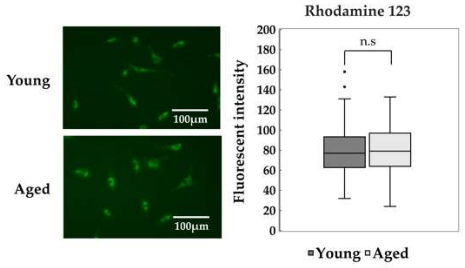

Supplementary Materials S2

Mitochondrial membrane potential in NHDFs stained with Rhodamine 123. Multiple fields of view of NHDF cells stained with Rhodamine 123 were observed. Representative images are shown. The scale bar indicates 100 μm. Fluorescence intensities of Young (104 cells) and Aged (91 cells) NHDFs obtained from three independent experiments (n=3) were compared using box-and-whisker plots. The boxes with quartiles shown in each section represent the median, 25th percentile, and 75th percentile. Whiskers indicate the maximum and minimum values within 1.5 times the interquartile range. Outliers are plotted separately. Dark boxes represent the Young NHDFs, while light boxes represent the Aged NHDFs. A Student's t-test was performed, and a significance level of $p < 0.05$ was considered statistically significant. The n.s indicates no significant difference.

Supplementary Materials. 3

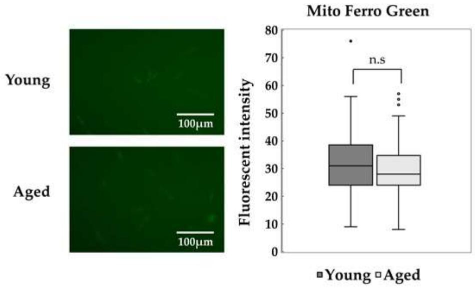

Supplementary Materials S3. **Free Fe^{2+} in Mitochondria of NHDFs**

Multiple fields of view in Mito Ferro Green-stained NHDF cells were observed. Representative images are shown. The scale bar indicates 100 μm. Fluorescence intensities in young (115 cells) and aged (102 cells) NHDFs obtained from three independent experiments (n=3) were compared using box-and-whisker plots. The boxes with quartiles shown in each section represent the median, 25th percentile, and 75th percentile. Whiskers indicate the maximum and minimum values within 1.5 times the interquartile range. Outliers are plotted separately. Dark boxes represent the Young NHDFs, while light boxes represent the Aged NHDFs. A Student's t-test was performed, and a significance level of $p < 0.05$ was considered statistically significant. The n.s indicates no significant difference.

Supplementary Materials. 4

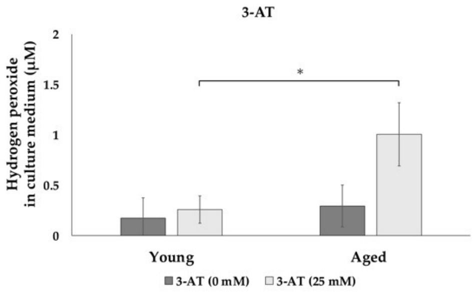

Supplementary Materials S4. **Hydrogen peroxide release from NHDFs inhibited by 3-AT**. Hydrogen peroxide concentrations secreted into the culture medium over 24 h from Young and Aged NHDFs were measured with or without CAT inhibition. CAT was inhibited by 3-AT (25 mM). Four independent measurements were performed (n=3), and the mean ± standard deviation is shown. A Student's t-test was performed, and a significance level of $p < 0.05$ was considered statistically significant. * indicates $p < 0.05$.

Supplementary Materials. 5

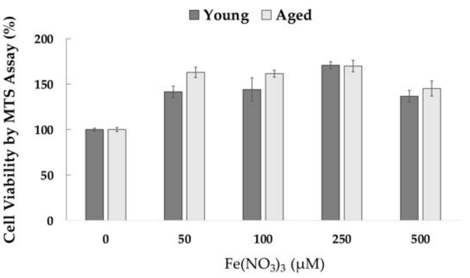

Supplementary Materials S5. **$Fe(NO_3)_3$-induced toxicity in NHDFs.** Cell viability (%) was assessed using the MTS assay for cells exposed to $Fe(NO_3)_3$ (0–500 μM) in FBS-free E-MEM for 24 h, and results are shown as mean ± standard deviation. ANOVA and Tukey's multiple comparison test were performed. A significance level of $p < 0.05$ was considered statistically significant. * indicate $p < 0.05$.

Supplementary Materials. 6

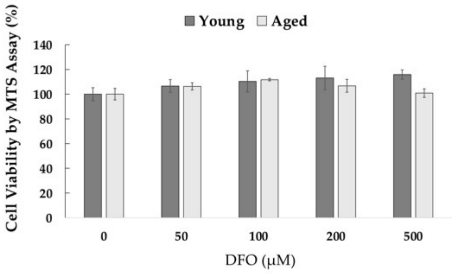

Supplementary Materials S6. **DFO-induced toxicity in NHDFs.** Cell viability (%) was assessed using the MTS assay for cells exposed to DFO (0–500 μM) in FBS-free E-MEM for 24 h, and results are shown as mean ± standard deviation. ANOVA and Tukey's multiple comparison test were performed. A significance level of $p < 0.05$ was considered statistically significant. * indicate $p < 0.05$.

Supplementary Materials. 7

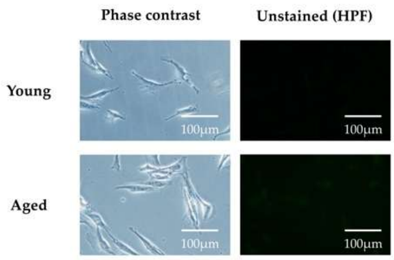

Supplementary Materials S7. **Negation of lipofuscin autofluorescence by unstained HPF.** No lipofuscin signal was observed in images captured under the same conditions as figure. 3(e).

Supplementary Materials. 8

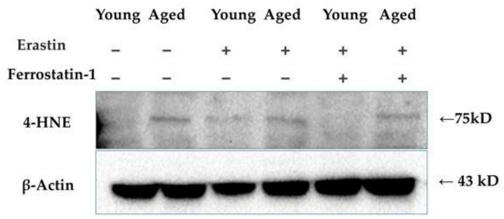

Supplementary Materials S8. **Detection of 4-HNE indicating lipid peroxidation.** NHDFs were incubated for 10 h in DMEM supplemented with either erastin (5 μM) or a mixture of erastin and Ferrostatin-1 (1 μM). Lysates were analyzed by Western blot. 15μg of protein per lane were loaded.
